# Increased intrinsic mitochondrial respiratory capacity in skeletal muscle from rats with streptozotocin-induced hyperglycemia

**DOI:** 10.14814/phy2.12467

**Published:** 2015-07-21

**Authors:** Steen Larsen, Celena Scheede-Bergdahl, Thomas Whitesell, Robert Boushel, Andreas Bergdahl

**Affiliations:** 1 Centre for Healthy Aging, Department of Biomedical Sciences, University of CopenhagenCopenhagen, Denmark; 2 Department of Kinesiology & Physical Education, McGill UniversityMontreal, Quebec, Canada; 3 Department of Exercise Science, Concordia UniversityMontreal, Quebec, Canada; 4 The Swedish School of Sport and Health SciencesStockholm, Sweden

**Keywords:** Hyperglycemia, mitochondria, OXPHOS, skeletal muscle, streptozotocin, type I diabetes

## Abstract

Type I diabetes mellitus (T1DM) is a chronic disorder, characterized by an almost or complete insulin deficiency. Widespread tissue dysfunction and deleterious diabetes-complications are associated with long-term elevations of blood glucose. The aim of this study was to investigate the effects of type I diabetes, as induced by streptozotocin, on the mitochondria in skeletal muscles that predominantly consist of either slow or fast twitch fibers. Soleus (primarily slow twitch fiber type) and the plantaris muscle (mainly fast twitch fiber type) were removed in order to measure mitochondrial protein expression and integrated mitochondrial respiratory function. Mitochondrial capacity for oxidative phosphorylation (OXPHOS) was found to be higher in the slow (more oxidative) soleus muscle from STZ rats when evaluating lipid and complex I linked OXPHOS capacity, whereas no difference was detected between the groups when evaluating the more physiological complex I and II linked OXPHOS capacity. These findings indicate that chronic hyperglycemia results in an elevated intrinsic mitochondrial respiratory capacity in both soleus and, at varying degree, plantaris muscle, findings that are consistent with human T1DM patients.

## New Findings

### What is the central question of this study?

The purpose of this study was to examine the effects of type 1 diabetes, as achieved by STZ, on skeletal muscle mitochondrial protein expression and integrated respiratory function in muscles with varying degrees of oxidative capacity.

### What is the main finding and its importance?

This study demonstrates that hyperglycemia, induced by STZ, results in an elevated intrinsic mitochondrial respiratory capacity in skeletal muscle when evaluating lipid and complex I linked substrates. This occurs in parallel with reduced mitochondrial content in both the slow, soleus and fast, plantaris muscle. These results help to further our understanding of the unique contributions of type 1 diabetes, and the associated lack of insulin, to the development and progression of diabetes-related complications.

## Introduction

Poorly controlled diabetes mellitus and resulting perturbations in blood glucose levels are implicated in widespread tissue dysfunction and deleterious organ complications (Blake and Trounce 2014). To better understand the development and progression of type I diabetes and its associated complications, an animal model has been established through the injection of streptozotocin (STZ). The administration of STZ leads to a rapid ablation of pancreatic *β*-cells, impairments in insulin production and subsequent hyperglycemia (King 2012). Hyperglycemia and the diminished effects, or complete lack, of insulin modify metabolic processes, thus contribute to the accelerated production of oxidative stress and irreversible metabolic by-products that pathologically alter cellular structure and function in many organs. These perturbations are directly implicated in the deleterious complications associated with the diabetic state (Brownlee 2001). For example, STZ-induced diabetes has been associated with myocardial contractile dysfunction that results from reductions in tricarboxylic acid (TCA) cycle enzyme levels, lower protein content of electron transport chain complex I and II and subsequent diminished mitochondrial oxidative phosphorylation (OXPHOS) capacity (Baseler et al. 2013). These impairments are causally linked to lipid peroxidation and carbonylation of antioxidative enzymes and protein structures (Baseler et al. 2013; Dennis et al. 2013). In skeletal muscle, protein expression and regulation of oxidative enzymes has been shown to be altered in the STZ model (Py et al. 2002; Choi et al. 2013). Increased oxidative stress in skeletal muscle, contributing to tissue dysfunction, has been linked to elevated expression and activity of xanthine oxidase and abnormal mitochondrial function in STZ-induced diabetes. Treatment of hyperglycemia by insulin reversed these pathological sources of oxidative stress (Bravard et al. 2011). Important for the understanding of these complications, it appears that the direct lack of insulin, and not necessarily the resulting hyperglycemia, negatively affects sensory neuron mitochondria and nerve conduction velocity, potentially playing a role in the development of neuropathy in the STZ rat model (Huang et al. 2003).

Why is it important to better understand the effects of STZ-induced diabetes on skeletal muscle? In skeletal muscle, glycolytic fibers appear to possess properties that potentiate the production and/or release of superoxide from the mitochondria, suggesting a mechanism for the development of heterogeneous mitochondrial dysfunction across tissue types (Anderson and Neufer 2006). STZ-induced diabetes negatively impacts the mitochondrial quality control system in skeletal muscle by means of the accumulation of proteins modified by reactive oxygen species (ROS) (Padrao et al. 2012). As in the case of sensory neurons previously mentioned, there appears to be a link between insulin deficiency and mitochondrial function. Franko and colleagues showed that, in murine STZ-induced diabetes, mitochondrial function was negatively impacted by means of a reduced transcription of mitochondrial genes mediated via decreased PGC-1*α* expression which was not evident in a type 2 or insulin-resistant model (Franko et al. 2012).

In light of the emerging role of the mitochondria in diabetes-related skeletal muscle dysfunction, the purpose of this study was to examine the effects of type 1 diabetes, as achieved by STZ, on skeletal muscle mitochondrial protein expression and integrated respiratory function in muscles with varying degrees of oxidative capacity. This information is important for the further understanding of the molecular mechanisms involved in the development of complications associated with insulin deficiency. We hypothesized that the mitochondria from the STZ muscles would experience reduced respiratory function compared with controls, in combination with a reduced mitochondrial content as evaluated by citrate synthase activity (CS). We further postulated that these differences would be greater in slow muscle fibers from soleus than in the fast muscle fibers found in plantaris, given their differences in oxidative capacities.

## Methods

### Animal care

Male Wistar rats (200–250 g) were obtained from Charles River Breeding Farms (St Constant, Quebec, Canada) and randomly assigned to either a control (CON) or a STZ group. Rats were housed individually in a thermo-neutral environment (22 degrees Celsius [°C]), on a 12:12 h photoperiod, and were provided access to standard dry laboratory rat chow and water ad libitum. All procedures were approved by the Animal Ethics Committee of Concordia University (BERG2010) and were conducted in accordance with guidelines of the Canadian Council on Animal Care.

### Induction of diabetes by STZ

To induce diabetes by STZ, each rat was temporary restrained using the Plas-Labs Flat-Bottom Rodent Restrainer from Fisher Scientific (Fisher Scientific, Ottawa, ON, Canada). Diabetes was brought on by a single bolus injection of STZ (65 mg/kg, Sigma, St. Louis, MO) into the tail vein (approximately 2 cm from base of tail) after site was wiped sterile with a mixture of 85% ethanol and 0.5% chlorhexidine. STZ was dissolved in less than 1 ml of 0.9% saline containing sodium citrate (20 mM/L, pH = 4.5) prior to injection. CON animals received a sham injection of citrate buffer only. All animals were closely monitored postinjection for adverse reactions not attributable to the effects of STZ. Diabetes was confirmed by low weight gain throughout the 4 week period as compared to CON, as well as high blood glucose (BG) concentrations. The weight as well as the blood glucose of the animals were logged every second day (Fig.2) using an Adventurer balance (OHAUS Corporation, Parsippany, NJ) and a Precision Xtra glucose meter (Abbot Laboratories, Mississauga, ON, Canada).

### Experimental protocol

The animals were euthanized by CO_2_, according to the approved animal ethics protocol. Both soleus (primarily type I fibers) and plantaris (mainly type II fibers) muscles were removed. One part of the muscle was snap frozen in liquid nitrogen, then stored at −80°C for biochemistry analysis, whereas the other muscle piece was placed in an ice-cold relaxing buffer (BIOPS) and used right away for the measurement of mitochondrial OXPHOS capacity.

### Preparation of permeabilized muscle fibers

Immediately after the muscles were removed from the rat, they were placed in an ice-cold buffer solution (BIOPS) containing (in mM): CaK_2_EGTA 2.77, K_2_EGTA 7.23, Na_2_ATP 5.77, MgCl_2_·6H_2_O 6.56, Taurine 20, Na_2_Phosphocreatine 15, Imidazole 20, Dithiothreitol 0.5, MES 50, pH 7.1. The muscle samples were gently dissected, connective tissue was removed and fiber bundles were separated using sharp forceps in ice-cold BIOPS buffer. After dissection, the fibers were incubated in 3 mL BIOPS buffer containing 50 *μ*g/mL saponin for 30 min. The fibers were then washed in ice-cold buffer (MiR05) for 2 × 10 min. MiR05 contains (in mM): EGTA 0.5, MgCl_2_·6H_2_O 3.0, K-lactonionate 60, Taurine 20, KH_2_PO_4_ 10, HEPES 20, Sucrose 110, BSA 1 g/l, pH 7.1. Measurements of oxygen consumption were performed at 37°C using high-resolution respirometry (Oxygraph-2k, Oroboros Instruments, Innsbruck, Austria). The respirometric measurements were performed in the buffer MiR05 loaded in each chamber. This technique has been described in further detail elsewhere (Boushel et al. 2007; Kuznetsov et al. 2008).

### Mitochondrial respiratory measurements

**Figure 1:**
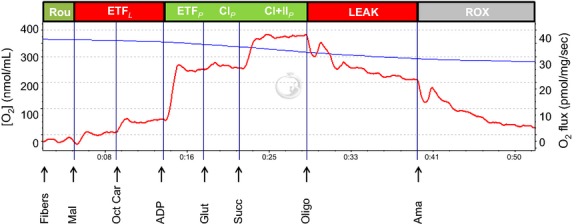
Representative trace of the respiratory flux from the substrate and inhibitor protocol. The blue line (*y*-axis, left) is oxygen concentration in the chamber. Red line (*y*-axis, right) is oxygen flux/mg tissue in the chamber. Addition of substrates and inhibitors as indicated on the figure. ADP, adenosine diphosphate; Ama, antimycin A; GLUT, glutamate; Mal, malate; Succ, succinate; Oct Car, octanoyl carnitine; Oligo, oligomycin.

The Oxygraph-2k respirometer was used in this study as it allows measurements with only 1.5–2.0 mg of muscle fibers (wet weight). Standardized calibrations were regularly performed (Gnaiger 2001). O_2_ flux was resolved by software (DatLab, Innsbruck, Austria), which converts nonlinear changes in the negative time derivative of the oxygen concentration signal. In order to avoid any potential oxygen limitation all experiments were carried out under hyperoxygenation conditions. All respirometric measurements were made simultaneously in duplicate. State 2 respiration (absence of adenylates) was assessed by addition of malate (2 mM) and octanoyl carnitine (1.5 mM; ETF_*L*_), state 3 respiration was achieved by adding ADP (5 mM; ETF_*P*_). This was followed by the addition of glutamate (10 mM; CI_*P*_) and succinate (10 mM; CI+II_*P*_), thus achieving maximal coupled respiration with convergent electron input to complex I and II of the electron transport system. Integrity of the outer mitochondrial membrane was tested by adding cytochrome *c* (10 *μ*M). If the respiration remained stable, the quality of the mitochondria was considered sufficient. Thereafter, oligomycin (2 *μ*g/mL) was added to block complex V (LEAK) followed by antimycin A (2.5 *μ*M) to inhibit complex III (ROX). Finally ascorbate (2 mM) and TMPD (500 *μ*M) were added to measure COX activity. A representative trace of the respiratory protocol, where the different states and titrations are shown can be seen in Figure1.

Citrate synthase (CS) activity was measured in a subgroup (*n* = 6) of the animals and mitochondrial OXPHOS capacity was normalized against the average of the measured CS activity, thus providing an indication of intrinsic mitochondrial function.

### Protein extraction, immunoblotting, and immunofluorescence

Cell lysates were extracted in lysis buffer containing (in mM) 250 NaCl, 50 HEPES (pH 7.5) 10% glycerol, 1% triton X-100, 1.5 MgCl_2_, 1 EGTA, 10 Na_4_P_2_O_7_, 1 NaF, 800 *μ*M Na_3_VO_4_ and centrifuged at 12,000 ×  *g* for 10 min. Supernatant was collected, and protein was measured using Pierce BCA Protein Assay Kit (Fisher Scientific, Ottawa, ON, Canada). 5 *μ*g of lysates was separated on a 10-12.5% SDS-PAGE and transferred to a nitrocellulose membrane (0.45 *μ*m, 162–0115, Bio-Rad) using 10 mM sodium tetraborate buffer. The membranes were blocked in 5% BSA in TBS-T buffer (10 mM Tris-HCl, pH 7.5, 150 mM NaCl, 0.05% Tween 20) for 1 h at room temperature followed by overnight incubation at 4°C with primary antibodies: Myosin I (1:3000, ab11083 Abcam), Myosin IIA (1:3000, ab24762 Abcam), Myosin IIB (1:3000, ab684 Abcam), Total OXPHOS Rodent antibody cocktail (1:500, MS604 MitoSciences). The blots were washed, incubated with horseradish peroxidase-conjugated secondary antibodies (anti-mouse, ab6728; anti-rabbit, ab6721; Abcam) and visualized with a chemiluminescence system (Immun-Star Chemiluminescent; 1705070; Bio-Rad). The bands were analyzed using Image J software.

### Citrate synthase activity

CS activity was determined in whole muscle homogenates using an assay kit (CS0720; Sigma-Aldrich). Total muscle protein was determined by the method of Pierce BCA Assay Kit, and the protein concentration of all samples equalized. CS activity was determined in triplicate based on the formation of TNB (2-nitro-5-thiobenzoic acid) at a wavelength of 412 nm at 25°C on a spectrophotometer (Biochrom WPA Biowave Spectrophotometer, Montreal Biotech, Montreal, Canada). In each well, 20 *μ*g of total protein sample was added to a reaction medium containing 178 *μ*L assay buffer, 2 *μ*L of 30 mm acetyl coenzyme A and 10 mm TNB acid. The baseline assay solution absorbance was recorded, reactions were initiated by addition of 10 *μ*L oxaloacetic acid, and the change in absorbance measured every 15 sec for 2 min. CS activity was only measured in a subgroup of the animals.

### Statistics

Data are presented as means ± SE in all figures and tables. For all statistical evaluations, *P *<* *0.05 was considered significant. One- and two-way ANOVAs with repeated measures for the time factor were performed. Significant main effects or interactions were further analyzed by the Holm–Sidak post hoc test. If the normality test failed, the data were log10 transformed and reanalyzed. The statistical analysis was performed using the software program SigmaStat 12.5 (Systat Software, San Jose, CA).

## Results

**Figure 2:**
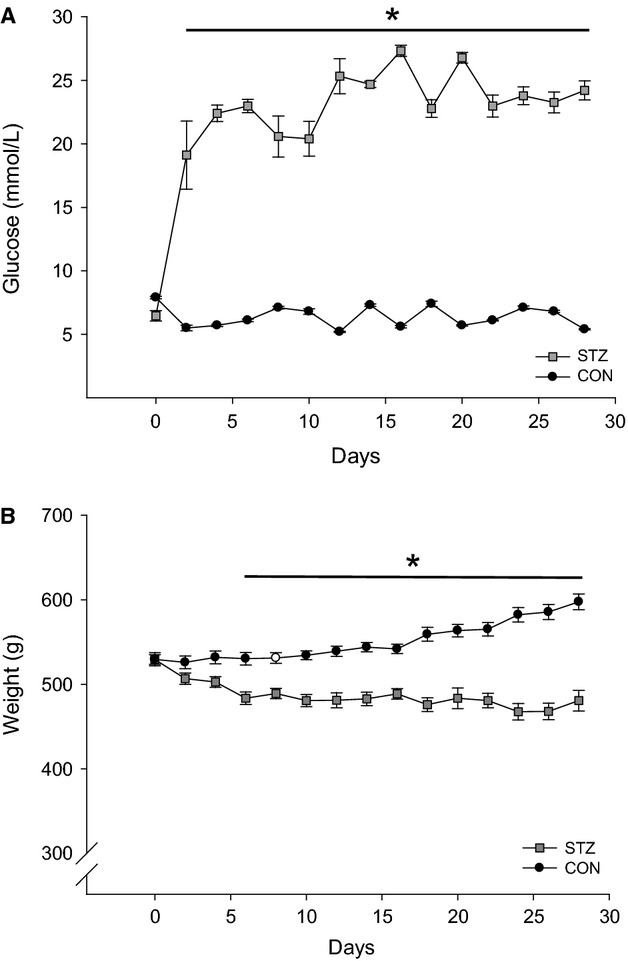
Glucose concentration and weight from baseline and during the 4 weeks after streptozotocin (STZ) administration (*n* = 10). Black circles represent control animals and gray squares represents STZ animals. (A) Glucose concentration. (B) Body weight. *(*P *<* *0.05) STZ versus control.

The STZ rats had increased blood glucose concentration after 4 weeks compared with CON. At the time of euthanasia, only the STZ rats had detectable ketone levels (data not shown) in the blood. The CON rats increased their weight during the 4 weeks compared with the STZ rats (Fig.2).

### Muscle protein characteristics measured by immunoblotting

**Figure 3:**
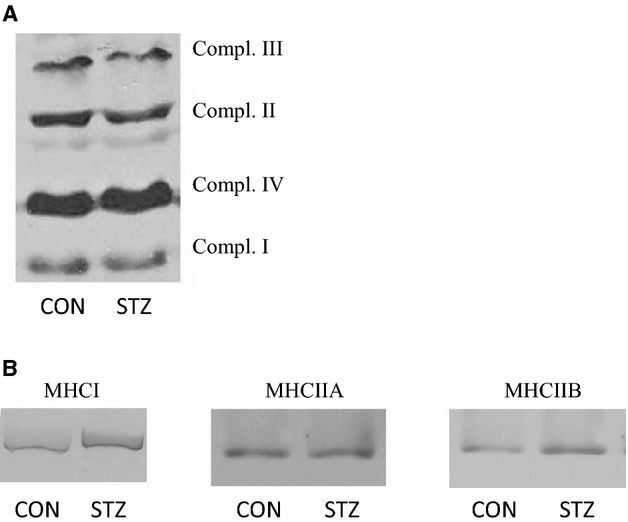
Representative gels for immunoblotting. (A) Mitochondrial subunits. (B) Myosin isoforms. For quantification the following bands were analyzed (in kDa): 20 (Complex I), 37 (Complex II), 48 (Complex III), 28 (Complex IV), 200 (MHCI), 226 (MHCIIA), and 240 (MHCIIB)

The relative amount of complex II was significantly reduced (*P *<* *0.05) in soleus from STZ compared with CON rats (Table1). No other differences were observed in complex I-IV content in either soleus or plantaris (Table1). Myosin Heavy Chain (MHC) composition was comparable in soleus between the groups, whereas STZ animals had an increased (*P *<* *0.05) abundance of the MHCI isoform in plantaris compared with CON animals (Table1). CS activity was significantly (*P *<* *0.05) higher in CON rats in both muscles compared with STZ rats (Table1). Representative gels are shown in Figure3.

**Table 1:** CS activity, complex I-IV content and MHC content in a subset of the animals (*n* = 6)

	Soleus		Plantaris	
	CON	STZ	CON	STZ
COMPLEX I (AU)	1.00 ± 0.03	0.89 ± 0.04	1.00 ± 0.16	1.05 ± 0.10
COMPLEX II (AU)	1.00 ± 0.05	0.68 ± 0.07*	1.00 ± 0.14	0.76 ± 0.19
COMPLEX III (AU)	1.00 ± 0.10	1.03 ± 0.03	1.00 ± 0.09	1.07 ± 0.11
COMPLEX IV (AU)	1.00 ± 0.09	0.95 ± 0.04	1.00 ± 0.07	0.82 ± 0.05
MHCI (AU)	1.00 ± 0.16	1.31 ± 0.11	1.00 ± 0.13	1.86 ± 0.25*
MHCIIA (AU)	1.00 ± 0.15	0.82 ± 0.07	1.00 ± 0.07	1.11 ± 0.10
MHCIIB (AU)	1.00 ± 0.04	0.99 ± 0.08	1.00 ± 0.24	1.26 ± 0.15
CS activity (*μ*moL/mL/min)	3.09 ± 0.31	2.32 ± 0.17*	2.29 ± 0.41	1.04 ± 0.31*

Protein measurements with mean values for CON set to 1.00, STZ compared to CON. Data are means ± SE.

AU, arbitrary units; CS, citrate synthase; MHC, myosin heavy chain

* (*P *<* *0.05) CON versus STZ.

### Mitochondrial respiration

**Figure 4:**
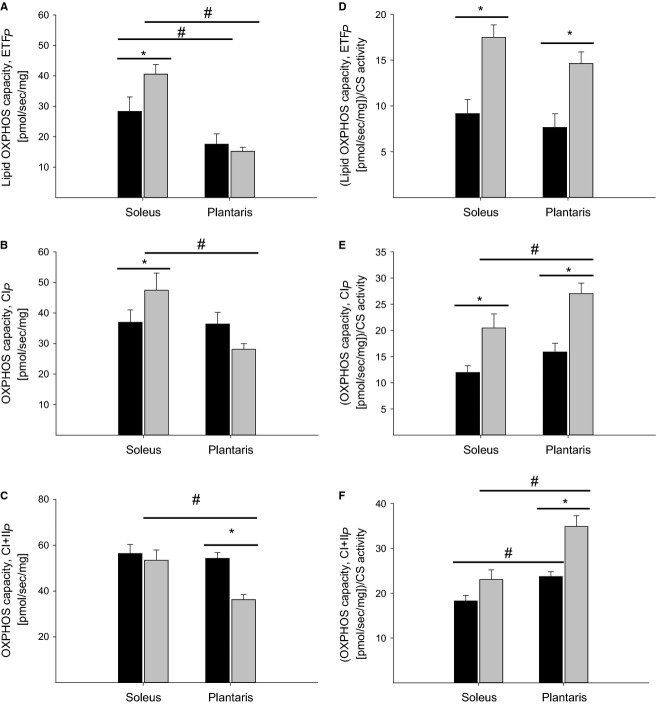
Mitochondrial OXPHOS capacity per mg tissue measured in permeabilized muscle fibers (*n* = 10). Black bar represents control animals and gray bar represents streptozotocin (STZ) animals. (A) Lipid OXPHOS capacity; (B) OXPHOS capacity with complex I linked substrates; (C) OXPHOS capacity with complex I and II linked substrates. (D–F) Are intrinsic respiratory capacity (mitochondrial OXPHOS capacity divided by CS activity), since CS activity was only measured in a subgroup (*n* = 8) of the animals, the average CS activity, was used to normalize mitochondrial OXPHOS capacity. Data are means ± SE. *(*P *<* *0.05) STZ versus control; ^#^(*P *<* *0.05) Soleus versus plantaris.

Lipid OXPHOS capacity was significantly higher (*P *<* *0.05) in soleus from STZ rats compared with CON rats (Fig.4A), whereas no difference was present in plantaris muscle. Both STZ and CON rats had a higher lipid OXPHOS capacity in soleus compared with plantaris muscle (Fig.4A). STZ rats showed a significantly higher OXPHOS capacity with complex I linked substrates in soleus compared with plantaris (Fig.4B). No difference was observed in OXPHOS capacity with complex I and II linked substrates in soleus muscle (Fig.4C), whereas plantaris muscle from STZ rats showed a significantly lower OXPHOS capacity (Fig.4C). No difference was found in complex IV OXPHOS capacity between STZ and CON rats or between the different muscles (data not shown). Residual oxygen consumption (ROX) was similar from STZ and CON rats in both soleus and plantaris (mean value), but was significantly lower in plantaris muscle from STZ rats compared with soleus muscle (data not shown). When mitochondrial OXPHOS capacity was normalized for mitochondrial content (CS activity), STZ rats showed a significantly higher (*P *<* *0.05) OXPHOS capacity per mitochondrion (intrinsic mitochondrial respiratory capacity) (Fig.4D–F) in both soleus and plantaris muscle compared with CON rats. Furthermore, intrinsic mitochondrial function was significantly higher (*P *<* *0.05) in plantaris compared with soleus in both STZ and CON rats with complex I + II linked substrates (Fig.4D–F).

**Figure 5:**
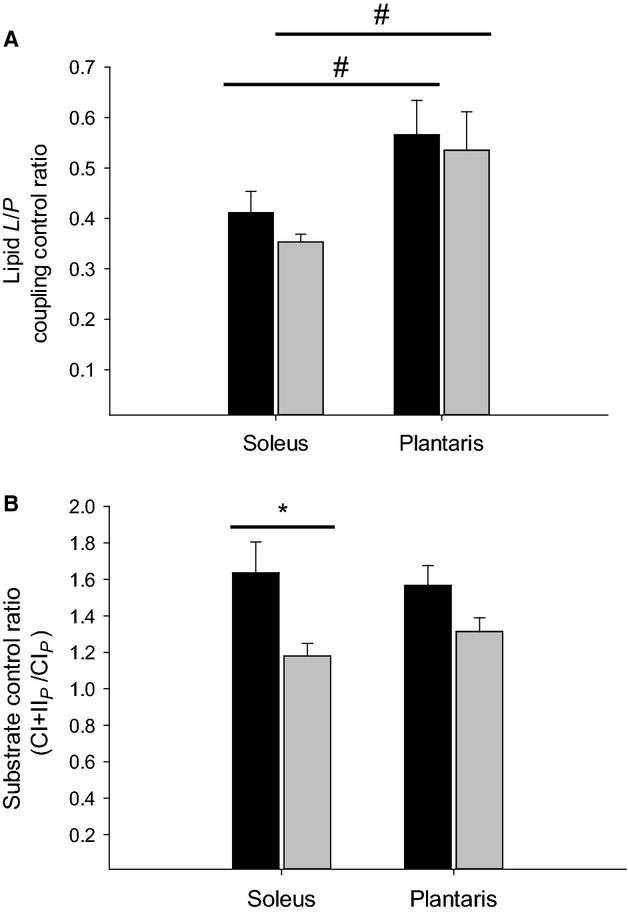
Black bar represents control animals and gray bar represents streptozotocin (STZ) animals. (A) Lipid coupling control ratio; (B) Substrate control ratio (SCR) (CI + II_*P*_/CI_*P*_). Data are means ± SE. *(*P *<* *0.05) STZ versus control; ^#^(*P *<* *0.05) Soleus versus plantaris.

Lipid coupling control ratio (L/P) was similar between STZ and CON rats in soleus as well as plantaris muscle, but was significantly higher in plantaris compared with soleus muscle from both STZ and CON rats (Fig.5A). Substrate control ratio for succinate (CI + II_*P*_/CI_*P*_) was significantly lower in soleus muscle from STZ rats compared to soleus muscle from CON rats (Fig.5B). No difference was seen in plantaris muscle, and no difference existed between the two different muscles.

## Discussion

The novel finding in this study is that mitochondrial OXPHOS capacity is higher in the oxidative soleus muscle from STZ rats when evaluating lipid and complex I linked substrate supply to the electron transfer system (Fig.4A and B), whereas no difference was detected between the groups when evaluating the more physiological complex I and II linked OXPHOS capacity (Fig.4C). These data indicate that complex II is impaired in STZ animals, which is supported by a reduced substrate control ratio (CI +I I_*P*_/CI_*P*_) and protein content of complex II. Furthermore, STZ-injected animals demonstrated a defect in the enzyme citrate synthase (CS) in both muscles analyzed. Therefore, when evaluating intrinsic mitochondrial respiration STZ rats exhibited higher O_2_ flux when compared with CON rats with lipid and complex I linked substrates (Fig.4A and B). No difference was present when complex I and II linked intrinsic capacity was evaluated in soleus muscle, but was significantly lower in plantaris muscle from STZ rats (Fig.4C).

Increased lipid oxidation in the rats injected with STZ, as we report here, is in agreement with human data from patients with insulin-dependent diabetes mellitus (T1DM), where a higher degree of fat oxidation has been reported (Raguso et al. 1995; Perseghin et al. 2005; Dumke et al. 2013). It has also been reported that STZ rats have lower total body lipid content (Rabinowitz and Craig 1989), which is consistent with data in human type 1 diabetes (Gomez et al. 2001). Whether these observations are due to the increased oxidation of lipids in type 1 diabetes is speculative. Interestingly, patients with type 2 diabetes are characterized by an unchanged or reduced fat oxidative capacity (Kelley and Simoneau 1994; Larsen et al. 2009). We also report that complex I linked respiration is increased in STZ soleus muscle. The increased lipid and complex I linked respiration could be a compensatory mechanism due to an impaired complex II linked respiration (Geromel et al. 1997). Whether this is a result of the lack of insulin remains to be further investigated.

Our findings also reveal there is no difference between STZ and CON when the mitochondria were maximally stimulated with ADP and complex I and II linked substrates in soleus muscle, but impaired in plantaris muscle. Those findings both imply that complex II linked respiration is impaired, which is matched by a reduced protein content of complex II in soleus muscle. This outcome is supported by previous work conducted in human subjects with magnetic resonance spectroscopy (Item et al. 2011), although the results are not consistent in the literature (Crowther et al. 2003). These two studies using magnetic resonance spectroscopy, do not clarify whether a defect is present in complex II. Our data could also be interpreted as STZ animals having a defect in CS. A decreased CS activity will lead to a reduced formation of citrate, which will result in an accumulation of oxaloacetate, which is known to be a potent inhibitor of complex II activity (Ackrell et al. 1974). This finding represents an important TCA cycle regulatory site, compounded by the reduction in complex II protein content. Citrate has been shown to play an intricate role in metabolic control through its interaction with other biochemical pathways especially phosphofructokinase to inhibit glycolysis (Iacobazzi and Infantino 2014). Citrate is a known chelator of divalent cations such as Fe^2+^ and thereby indirectly interferes with the Fenton reaction between iron and hydrogen peroxide, likely effecting the production of hydroxyl radicals. Interestingly, it has been shown that the reactivation of aconitase after oxidative stress is fully dependent on the presence of citrate through an interaction with frataxin (Bulteau et al. 2004). It can be speculated that a reduction in citrate formation results in a functional loss of protection of the muscle cell against oxidative stress. Increased levels of ROS (H_2_O_2_) have been reported to inhibit complex II linked respiration and complex II activity in isolated rat cardiac mitochondria (Moser et al. 2009). Hyperglycemia elevates mitochondrial-induced oxidative stress in human type 1 and experimental diabetes (Baynes 1991; Wentholt et al. 2008). Changes in substrate availability, impaired function of respiratory complexes and increased mitochondrial leak may predispose cells to elevated production of reactive oxygen species and subsequent apoptosis (Williamson et al. 2010; Bravard et al. 2011). The elevated intrinsic mitochondrial function seen in STZ rats, could indeed result in a higher membrane potential and ROS production. It has been reported that ROS production is higher in STZ compared to CON mice (Bravard et al. 2011). Further studies are needed to elucidate the relationship between ROS production in combination with the evaluation of mitochondrial OXPHOS capacity.

As STZ is a model of type 1 diabetes, our findings imply that insulin action in skeletal muscle couples directly to mitochondrial energetics and substrate selection. A lower OXPHOS capacity was observed in plantaris muscle from STZ rats when complex I and II linked substrates were used (Fig.4C), whereas no detectable differences were seen between the groups with the other substrate combinations (Fig.4A and B). When intrinsic mitochondrial respiratory capacity was evaluated, MHC-1 content should equate with a higher mitochondrial density, but this was not the case in this study where a reduced CS activity was seen in the plantaris muscle from the STZ rats. Our findings are supported by previous work conducted in mice, where an increased MHC-1 content was accompanied by a reduced mitochondrial content (as evaluated by succinate dehydrogenase activity) (Eshima et al. 2013). Other studies have also reported a reduction in mitochondrial density in skeletal muscle from animals with STZ-induced diabetes and this is consistent with our results that indicate impaired CS activity and a reduced complex II protein content in the STZ rats (Py et al. 2002; Bravard et al. 2011; Padrao et al. 2012).

We also report that intrinsic mitochondrial function is elevated in STZ rats in both muscle types. CS activity was only measured in a subgroup of the animals (*n* = 6), and the average CS activity was used to calculate intrinsic mitochondrial function. These results differ from previous findings obtained from patients with type 2 diabetes, where a reduced intrinsic mitochondrial respiratory capacity was observed (Mogensen et al. 2007). These inconsistencies may be due to the extent and duration of hyperglycemia as patients with type 2 diabetes may remain undiagnosed and therefore untreated for years, whereas the rats in this study were only diabetic for a period of 4 weeks. Elevated intrinsic mitochondrial function may be due to a compensatory effect, although this remains speculative. Insulin treatment has in humans been shown to decrease mitochondrial proton leak, but the effects of insulin on mitochondrial function are also not well understood and remain to be elucidated (Rabøl et al. 2009).

In conclusion this study demonstrates that hyperglycemia, induced by STZ, results in an elevated intrinsic mitochondrial respiratory capacity in skeletal muscle. Furthermore, this occurs in muscle tissue of varying oxidative capacity as demonstrated in both soleus and plantaris muscle. This elevated intrinsic capacity in STZ-treated rats is consistent with human patients with type 1 diabetes. We propose that the higher intrinsic respiratory capacity may be linked to an increased production of reactive oxygen species in muscle, which could potentially result in apoptosis. Taken together our data suggest that increased intrinsic mitochondrial respiratory capacity could be an early event in the pathogenesis of type 1 diabetes. Similar studies in human type 1 diabetes are warranted as the development and progression of complications associated with this disease have been linked to these mitochondrial abnormalities.
